# Peripheral Nerve Transplantation Combined with Acidic Fibroblast Growth Factor and Chondroitinase Induces Regeneration and Improves Urinary Function in Complete Spinal Cord Transected Adult Mice

**DOI:** 10.1371/journal.pone.0139335

**Published:** 2015-10-01

**Authors:** Marc A. DePaul, Ching-Yi Lin, Jerry Silver, Yu-Shang Lee

**Affiliations:** 1 Department of Neurosciences, Case Western Reserve University, Cleveland, Ohio, United States of America; 2 Department of Neurosciences, Lerner Research Institute, Cleveland Clinic, Cleveland, Ohio, United States of America; Hertie Institute for Clinical Brain Research, University of Tuebingen., GERMANY

## Abstract

The loss of lower urinary tract (LUT) control is a ubiquitous consequence of a complete spinal cord injury, attributed to a lack of regeneration of supraspinal pathways controlling the bladder. Previous work in our lab has utilized a combinatorial therapy of peripheral nerve autografts (PNG), acidic fibroblast growth factor (aFGF), and chondroitinase ABC (ChABC) to treat a complete T8 spinal cord transection in the adult rat, resulting in supraspinal control of bladder function. In the present study we extended these findings by examining the use of the combinatorial PNG+aFGF+ChABC treatment in a T8 transected mouse model, which more closely models human urinary deficits following spinal cord injury. Cystometry analysis and external urethral sphincter electromyograms reveal that treatment with PNG+aFGF+ChABC reduced bladder weight, improved bladder and external urethral sphincter histology, and significantly enhanced LUT function, resulting in more efficient voiding. Treated mice’s injured spinal cord also showed a reduction in collagen scaring, and regeneration of serotonergic and tyrosine hydroxylase-positive axons across the lesion and into the distal spinal cord. Regeneration of serotonin axons correlated with LUT recovery. These results suggest that our mouse model of LUT dysfunction recapitulates the results found in the rat model and may be used to further investigate genetic contributions to regeneration failure.

## Introduction

Axonal regeneration following spinal cord injury (SCI) in the adult is limited and abortive. Regeneration failure has been attributed to many factors including myelin inhibition [1], extracellular matrix-associated inhibitors [2], a decrease of intrinsic growth-promoting gene expression [3,4], secondary injury cascades involving the immune response [5], and lack of trophic support [6]. Targeting a single factor can increase axonal sparing, regeneration, and/or sprouting [7] into or around the lesion site, but the extent of growth is modest. Instead, an approach combining several targets can act synergistically to promote robust regeneration [8–10]. For example, using peripheral nerve autografts (PNGs) to bridge a complete spinal cord lesion in combination with acidic fibroblast growth factor (aFGF) and chondroitinase ABC (ChABC) leads to long-distance axonal regeneration and restores supraspinal control of bladder function in a complete transection rat SCI. Removing even one factor from this approach diminishes recovery and axonal regeneration [8]. While most complex combinatorial approaches have been conducted in rats, mice can offer greater insight into genetic contributions to SCI failure [3,4,11]. Mouse studies often target a single gene or a related family of genes and have shown great promise in promoting regeneration but, thus far, have resulted in only minimal recovery [12,13].

A robust and evolutionally conserved mechanism of SCI therapy should effectively treat injuries in any mammal, even when the pathology and recovery can be vastly different from species to species. Closely related organisms such as the rat and mouse can differ drastically in response to SCI. In rats, a fluid-filled cystic cavity forms at the site of injury, while the injured mouse spinal cord becomes filled with fibrous connective tissue and a dense distribution of collagen scarring [14,15]. The inflammatory response is distinct between the two species, leading to different cell populations in and around the lesion [16]. Physiological recovery differences after severe SCI also exist. Partial recovery of the lower urinary tract (LUT) is spontaneous in the rat [17–19], while mice never regain the ability to urinate after severe SCI [15]. Under normal physiological conditions, external urethral sphincter (EUS) muscle recruitment differs between rats and mice. In rats, EUS pumping activity causes high frequency oscillations in bladder pressure, and is necessary for voiding, however, EUS bursting coupled with high frequency bladder pressure oscillations are not observed in the mouse during a void. Instead, the mouse EUS displays silent periods or periods of low EUS activity that lack bladder pressure oscillations, similar to what is seen in humans [20]. Ideally, animal models should reproduce all the facets of the human condition, but it is inconceivable that any single animal model will recapitulate every aspect of normal or SCI pathology. For these reasons, a mouse model of SCI may better represent bladder dysfunction in the human condition, as the loss of LUT control is a permanent ubiquitous consequence of SCI in humans and is a top priority of the SCI population [21,22].

In this present study we used a well-documented SCI combinatorial repair strategy first developed in a rat model and now adopted to a mouse model [8,23–25]. Specifically, we used multiple PNGs covered by an aFGF-laden fibrin matrix plus ChABC and aFGF delivered to the graft and graft/host interfaces to create an environment favorable for regeneration that, for the first time in a complete transection mouse model, demonstrated improvements in LUT function.

## Materials and Methods

### Animal groups

Thirty-seven adult female C57BL/6 mice (8 to 10 weeks old) were divided randomly into three groups: (1) T8 spinal cord transection only (Tx-only; n = 15), (2) T8 spinal cord transection with PNG+aFGF+ChABC treatment (PNG+aFGF+ChABC; n = 15), (3) naïve animal without SCI (n = 7). Five mice were removed from the study (three from the PNG+aFGF+ChABC group and two from the TX-only group) due to bladder infections and/or bladder stones and were not included in any analyses.

### Mouse spinal cord surgery, multiple peripheral nerve segment transplantation, and ChABC & aFGF injection

All sterile surgical procedures were carried out in strict accordance with the recommendations in the Guide for the Care and Use of Laboratory Animals of the National Institutes of Health. The protocol was approved by the Case Western Reserve University animal resource center and institutional care and use committee. Surgery was performed under 2% isoflurane mixed with oxygen anesthesia, and all efforts were made to minimize suffering. Mice were supported on a heating pad controlled by a rectal thermometer maintained within 1.5°C of normal temperature. In all SCI groups, the thoracic spinal cord was exposed via T8 laminectomy. In the TX-only group the spinal cord was transected by two parallel transverse cuts, creating a 0.5 mm gap when the tissue was removed. The PNG+aFGF+ChABC group underwent a spinal cord transection as described above and 2 μl (1 μl at each cord stump adjacent to the lesion) of 1:1 ChABC (1U/ml) & aFGF (1 g) mixture was injected into the spinal cord via a Nanoject II (Drummond Scientific Company). The intercostal nerves were removed, soaked in ChABC (1U/ml) for 30 min, and an autograft was constructed from 9–15 intercostal nerve segments spanning the lesion, as described previously in rat [24]. The graft was supported using an aFGF/ChABC/fibrin glue. 4–0 monofilament sutures were used to close the skin and musculature. Bladders were manually expressed twice per day until the end of the study.

### Urodynamics/cystometrogram and electromyography recordings

Mice at 18 weeks post-SCI were anesthetized with 1.5% isoflurane mixed with oxygen. A polyethylene-50 catheter was carefully inserted through the urethra into the bladder for the delivery of saline and bladder pressure monitoring. Teflon-insulated silver wire electrodes (0.003” diameter, 2 mm exposed tip; A-M Systems) were inserted percutaneously via the vagina on both sides of the urethra to monitor EUS electromyography (EMG) activity. The mice were placed in a restraining apparatus and allowed to recover from anesthesia. Bladders were emptied prior to the start of saline infusion. Continuous cystometrograms (CMGs) were collected using constant infusion (1.5 ml/hr) of room temperature saline (Aladdin-1000 single syringe infusion pump; World Precision Instruments) through the catheter and into the bladder to elicit repetitive voids, which allowed collection of data for a large number of voiding cycles. The electrodes were connected to a preamplifier (HZP; Grass-Technologies), which was connected to an amplifier (QP511, Grass-Technologies) with high- and low-pass frequency filters at 30 Hz and 3 kHz and a recording system (Power 1401, Spike2; Cambridge Electronic Design) at a sample frequency of 10kHz. The bladder pressure was recorded via the same catheter used for saline infusion, using a pressure transducer (P11T, Grass-Technologies) connected to the recording system at a sample frequency of 2kHz.

### Urodynamic quantification

Mice began infusion of saline with an empty bladder. The time point where the first void occurred was used to calculate the bladder volume at first void (time x rate of infusion). Continuous saline infusion elicited repetitive voiding. The bladder contraction interval was determined by measuring the average time between bladder contractions. The change in pressure during void was measured as the bladder pressure at the peak of a void minus the pressure immediately prior to the void. The pressure difference from baseline to post-void was measured as the lowest pressure recorded after the first void minus the pressure at the start of saline infusion. Following the last void the catheter was removed and residual bladder saline was expressed to measure residual volume.

### Bladder weight and morphology

Animals were perfused transcardially with 4% paraformaldehyde (PFA) in 0.01 M PBS, pH 7.4. The bladders of all animals were collected and the wet weights were recorded. Bladders were further fixed with 4% PFA for 1 day and then transferred to 30% sucrose, sectioned transversely (8 μm) at the level of mid-bladder with a cryostat, and stained with Masson’s trichrome for additional morphological analysis under a light microscope.

### Spinal cord histology

Following perfusion, spinal cords from all animals were collected and immersed in a 30% sucrose solution. Sagittal sections (30 m thick for immune staining, 10 μm thick for Masson’s trichrome staining) of the spinal cord were cut using a cryostat and collected for immunostaining or Masson’s trichrome staining. For immunostaining, sections were blocked in 3% normal horse serum with 0.25% Triton X-100 in PBS for 1 hour. After blocking, sections were exposed to anti-tyrosine hydroxylase (TH) polyclonal antibody (1:1000 dilution, Protos Biotechnology) or anti-serotonin (5-HT) polyclonal antibody (1:1500 dilution; DiaSorin, Stillwater, MN, USA), and anti-glial fibrillary acidic protein (GFAP) for astrocytes (1:500 dilution; DakoCytomation, Carpinteria, CA, USA) and incubated overnight at room temperature. After 3 rinses in PBS, sections were incubated with species-appropriate secondary antisera conjugated with Alexa Fluor 594 or Alexa Fluor 488 (Invitrogen/Molecular Probes) for 90 min, washed, and coverslipped with Vectashield (Vector Laboratories Inc., Burlingame, CA, USA). All sections were examined under a microscope with fluorescent light and the distribution of TH and 5-HT fibers was analyzed in multiple parallel sections using a camera lucida method. Further images were collected using a Carl Zeiss LSM 510META confocal microscope. Masson’s trichrome staining of the spinal cord was conducted in the same manner as the bladder. For collagen quantification, sections were imaged under a light microscope. For each animal, the area 500μm rostral and 500μm caudal from the lesion epicenter were quantified and averaged over 5 serial sections to obtain a single measurement per animal of the collagen area squared.

### Statistical analyses

All data are reported as mean ± standard error of the mean. All data sets except for collagen quantification were analyzed using Unpaired Student's T tests comparing TX-only to PNG+aFGF+ChABC groups. Collagen quantification was analyzed using Two-way ANOVA. Significance was determined at p < 0.05. Naïve animals are included for comparison only and were not included in the statistics since they did not receive an injury. 5-HT and TH correlation to bladder recovery was determined using Pearson product-moment correlation coefficient. All behavior tests and data analysis were done in a blinded fashion during this entire study.

## Results

### PNG + aFGF + ChABC improved and EUS EMG activity in spinal cord-transected mice

**Fig 1:**
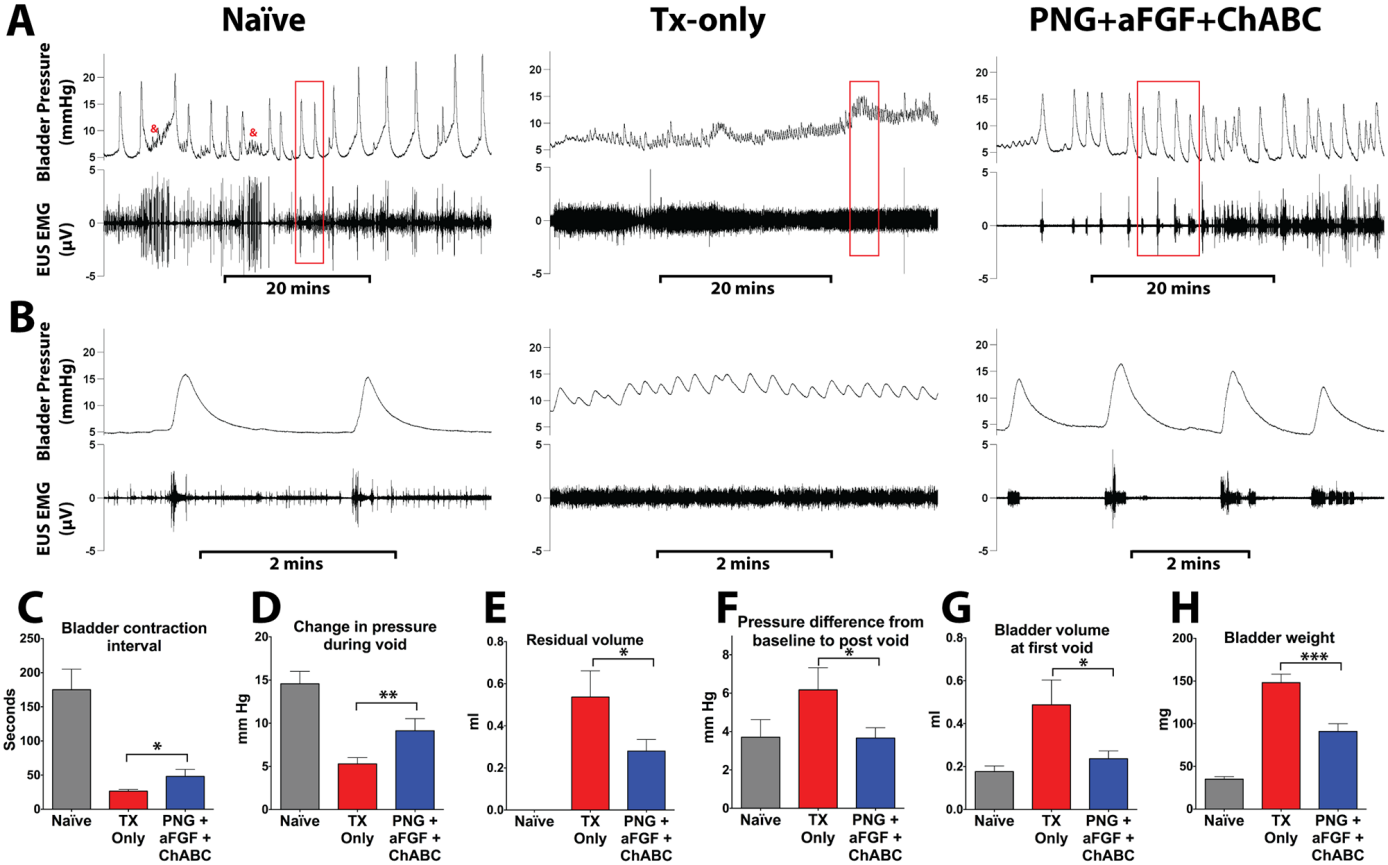
PNG+aFGF+ChABC treatment improves urodynamics after complete SCI. (A) Representative voiding cycles of bladder pressure (top panel) and EUS EMG activity (bottom panel) were recorded from each group 18 weeks post-complete spinal cord transection. Kicking artifacts in naïve bladder tracings are denoted by &. (B) The box area in (A) indicates the magnification of bladder pressure and EUS EMG activity. Quantification of CMG results shows that PNG+aFGF+ChABC-treated animals (C) increased the time between bladder contractions, (D) had stronger bladder contractions during voids, (E) had a smaller residual volume after a void, (F) that pressure following a void was closer to baseline pressure, (G) voided at smaller bladder volumes, and (H) had a smaller bladder weight when compared to TX-only. *p<0.05, **p<0.01, ***p < .001.

Measurements of bladder contractions and EUS activity were used to investigate the quality of bladder function at 18 weeks after SCI. Recordings were performed on awake and restrained animals. Movement artifacts in naïve recordings were evident and frequent (Naïve, Fig 1A) but could easily be separated from relevant events. Minimal movement artifacts were seen in spinalized mice. Thirteen animals in the Tx–only group, twelve in the PNG+aFGF+ChABC group, and seven in the naïve group were used in this investigation.

During continuous infusion CMG, the naïve group displayed low-amplitude tonic EUS-EMG activity between voids (Naïve, Fig 1A and 1B). At the onset of a bladder contraction, the EUS-EMG activity increased in amplitude and persisted as the pressure rose, falling at the peak of the contraction coinciding with a void (Naïve, Fig 1B). In transected mice, the coordination between rising bladder pressure and EUS activity was lost and the EUS fired tonically throughout the bladder contraction (TX-only, Fig 1A and 1B), resulting in severe detrusor sphincter dyssynergia. Animals receiving PNG+aFGF+ChABC showed an increase in EUS-EMG activity in response to rising bladder pressure and an improvement in bladder and EUS coordination, similar to what was seen in the naïve group (PNG+aFGF+ChABC, Fig 1A and 1B).

In the naïve group, bladder contractions were associated with voiding events. The few bladder contractions outside of voiding events were associated with movement within the restrainer (Naïve Fig 1A). At eight weeks post-SCI, there was no difference in CMG-EUS EMG data between the TX-only and PNG+aFGF+ChABC groups (data not shown). However, at 18 weeks post-SCI, the TX-only group had many non-voiding contractions, and in many recordings voiding and non-voiding contractions could not be distinguished. These contractions were typically very small and close together (TX-only Fig 1A). In contrast, bladder contractions in the PNG+aFGF+ChABC group at 18 weeks post-SCI were often associated with a voiding event, although they still showed signs of a hyperactive bladder (PNG+aFGF+ChABC, Fig 1A). The time between bladder contractions increased in the PNG+aFGF+ChABC group (48.07 ± 10.32 seconds) compared to the TX-only group (26.54 ± 2.33, p < 0.05, Fig 1C), suggesting that the treatment alleviated symptoms of bladder hyperactivity.

Bladder contractions resulting in a void occurred at similar pressures between the spinal cord-injured groups (TX-only = 11.56 ± 1.16 mmHg; PNG+aFGF+ChABC = 9.80 ± 0.72, p = 0.109 mmHg) but the pressure generated by the PNG+aFGF+ChABC group was significantly larger (9.14 ± 1.40 mmHg) than in TX-only (5.29 ± 0.72 mmHg, p < 0.01, Fig 1D). The pressure following a void also returned closer to the baseline in the combinatorial group (PNG+aFGF+ChABC = 3.66 ± 0.54 mmHg above baseline, TX-only = 6.17 ± 1.17 mmHg above baseline, p < 0.05, Fig 1E), resulting in more efficient voiding and a smaller residual volume (PNG+aFGF+ChABC = 0.28 ± 0.06 ml; TX-only = 0.54 ± 0.12 ml, p < 0.05, Fig 1F). The CMG analysis also showed that the first void of the PNG+aFGF+ChABC group occurred at a smaller volume (0.16 ± 0.02 ml) compared to TX-only (0.33 ± 0.08 ml, p < 0.05, Fig 1G). Together, the CMG data indicate that mice treated with a combination of PNG+aFGF+ChABC show significant improvements in bladder voiding and a reduction in bladder hyperreflexia.

### PNG+aFGF+ChABC treatment improved LUT morphology

**Fig 2:**
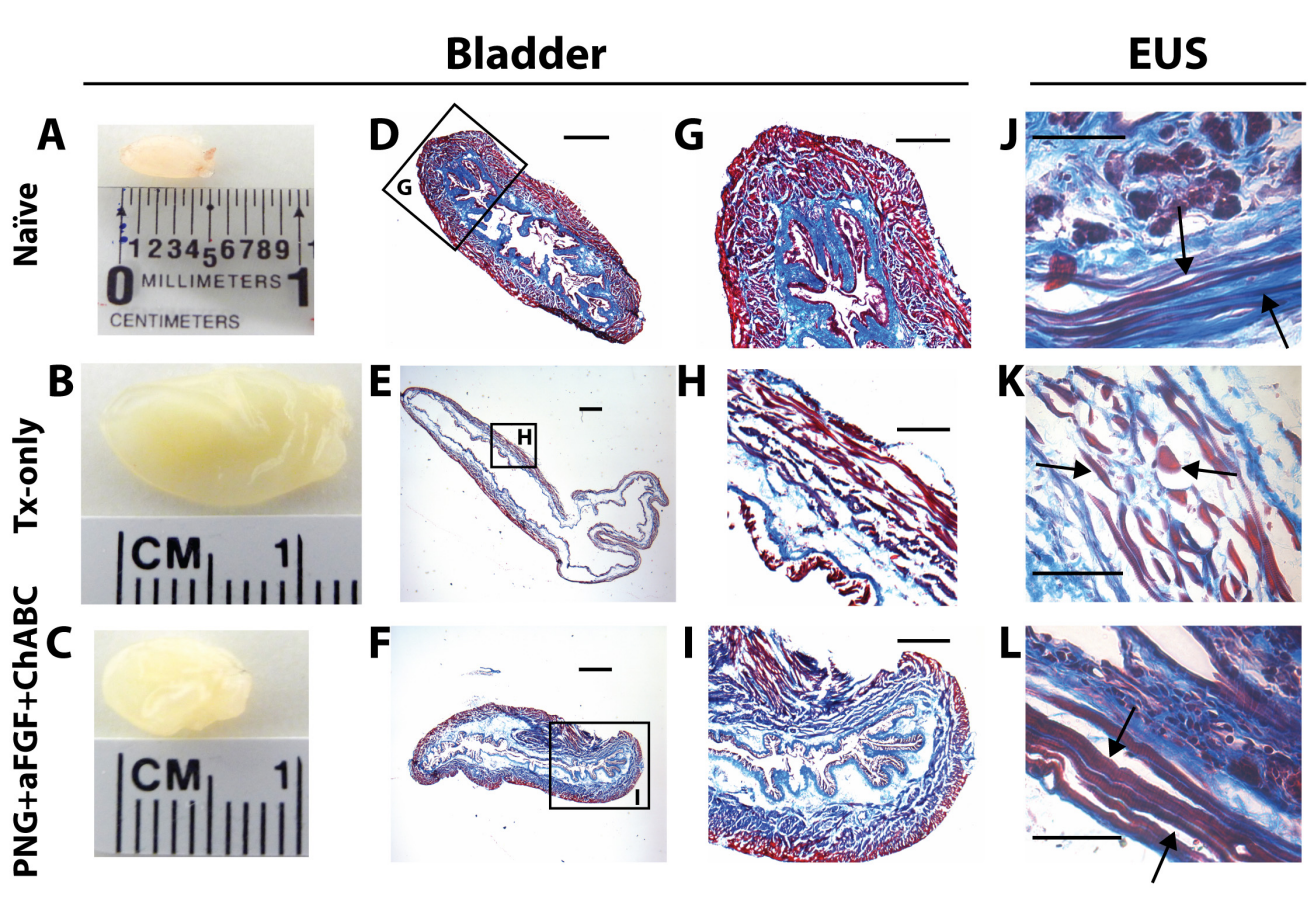
PNG+aFGF+ChABC treatment improves bladder and EUS morphology. (A-C) Gross observation of the bladder at 18 weeks after SCI in naïve (A), TX-only (B), and PNG+aFGF+ChABC animals (C). (D-F) Photomicrographs of transverse bladder sections stained with Masson’s trichrome from naïve (D), TX-only (E), and PNG+aFGF+ChABC animals (F). Scale bar, 500μm. (G-I) Higher magnification of photomicrographs from boxed area (D-F) shows details of the bladder structure. Note that PNG+aFGF+ChABC animals showed improved bladder morphology. Sale bar, 250μm. (J-L) Photomicrographs of transverse EUS sections stained with Masson’s trichrome from naïve(J), TX-only (K), and PNG+aFGF+ChABC animals (L). The TX-only animals show swelling as well as short and discontinuous muscle fibers (arrow), while the PNG+aFGF+ChABC animals show continuous and long muscle fibers (arrow), similar in morphology to naïve animals (arrow). Scale bar, 500μm.

We next examined the anatomical changes that may underlie the improvements seen in LUT physiology. Comparing gross observations of the bladder 18 weeks after SCI, mice in the PNG+aFGF+ChABC group had smaller bladders that weighed significantly less than those in the TX-only group (Figs 1H,2B and 2C), although both injury groups had larger and heavier bladders than naïve mice (Figs 1H and 2A). Masson's trichrome stain of the bladder revealed preserved architecture and less bladder distension with greater organization of the muscle and lamina propria in PNG+aFGF+ChABC animals compared to the TX-only group (Fig 2D–2I).

EUS muscle fibers of the PNG+aFGF+ChABC group were long and continuous (Fig 2L), similar to the naïve group (Fig 2J), while the TX-only group had a greater occurrence of connective tissue between short and discontinuous muscle fibers (Fig 2K). The denervation of motor neurons controlling EUS causes tonic activity of EUS after complete SCI. The constant tonic activity of EUS and severe detrusor sphincter dyssynergia may contribute to the muscle loss and degradation. These results suggest preservation of muscle and architecture in the LUT with PNG+aFGF+ChABC treatment.

### PNG+aFGF+ChABC treatment reduces collagen scarring

**Fig 3:**
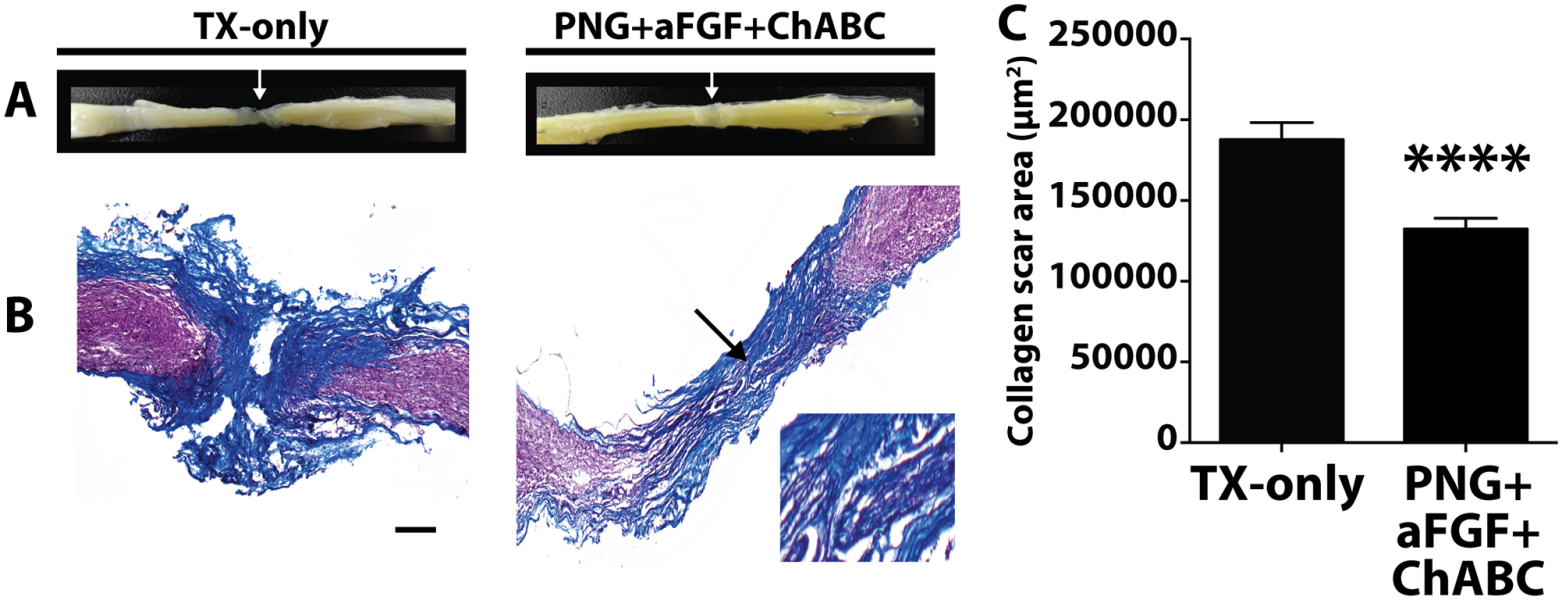
PNG+aFGF+ChABC decreases collagen scaring in the lesion. (A) Gross observation of the spinal cord at 18 weeks after SCI in TX-only and PNG+aFGF+ChABC animals. White arrow marks the lesion. (B) Representative photomicrographs of the spinal cord lesion stained with Masson’s trichrome from TX-only and PNG+aFGF+ChABC mice 18 weeks post injury. The morphology of the PNG can be visualized in the PNG+aFGF+ChABC treated animals. Arrow marks the PNG and area of expanded view. Scale bar, 200μm. Rostral is to the left, and caudal to the right. Collagen is stained blue. (C) Collagen scar area (μm^2^). The PNG+aFGF+ChABC group shows a significant reduction of collagen scarring compared to the TX-only group. Five serial sections per animal were quantified, n = 5 animals per group. ****p<0.0001.

We next examined the lesion of the TX-only and PNG+aFGF+ChABC for differences in gross anatomy and scarring. In the TX-only group, the rostral and caudal stumps of the lesioned cord were connected by thin, translucent tissue containing a dense collagen matrix (Fig 3A and 3B). The distribution of collagen dense deposits can be observed in the lesion gap, in the periphery around the cord, and inside the spinal cord tissue from both rostral and caudal end. In contrast, the lesioned cord of animals receiving PNG+aFGF+ChABC treatment were thick and opaque with dense peripheral nerve grafts bridging the rostral and caudal stumps completely filling the lesion, giving an appearing of a continuous interface across the lesion (Fig 3A). The collagen matrix of PNG+aFGF+ChABC treated animals was less dense and covered significantly less area than that of the TX-only treatment (Fig 3B and 3C). To compare the mouse response to injury to that of the rat, we collected available complete spinal cord transection tissue in our laboratory and stained for collagen. The rat lesion had very little collagen scarring compared to the mouse, illustrating the difference in scar composition between the two species (S1 Fig). These results suggest that PNG+aFGF+ChABC treatment can significantly reduce collagen scarring in the mouse, which can facilitate axonal regeneration by decreasing the physical scar barrier.

### Regeneration of 5-HT and TH fibers crossing the PNG into the caudal spinal cord

**Fig 4:**
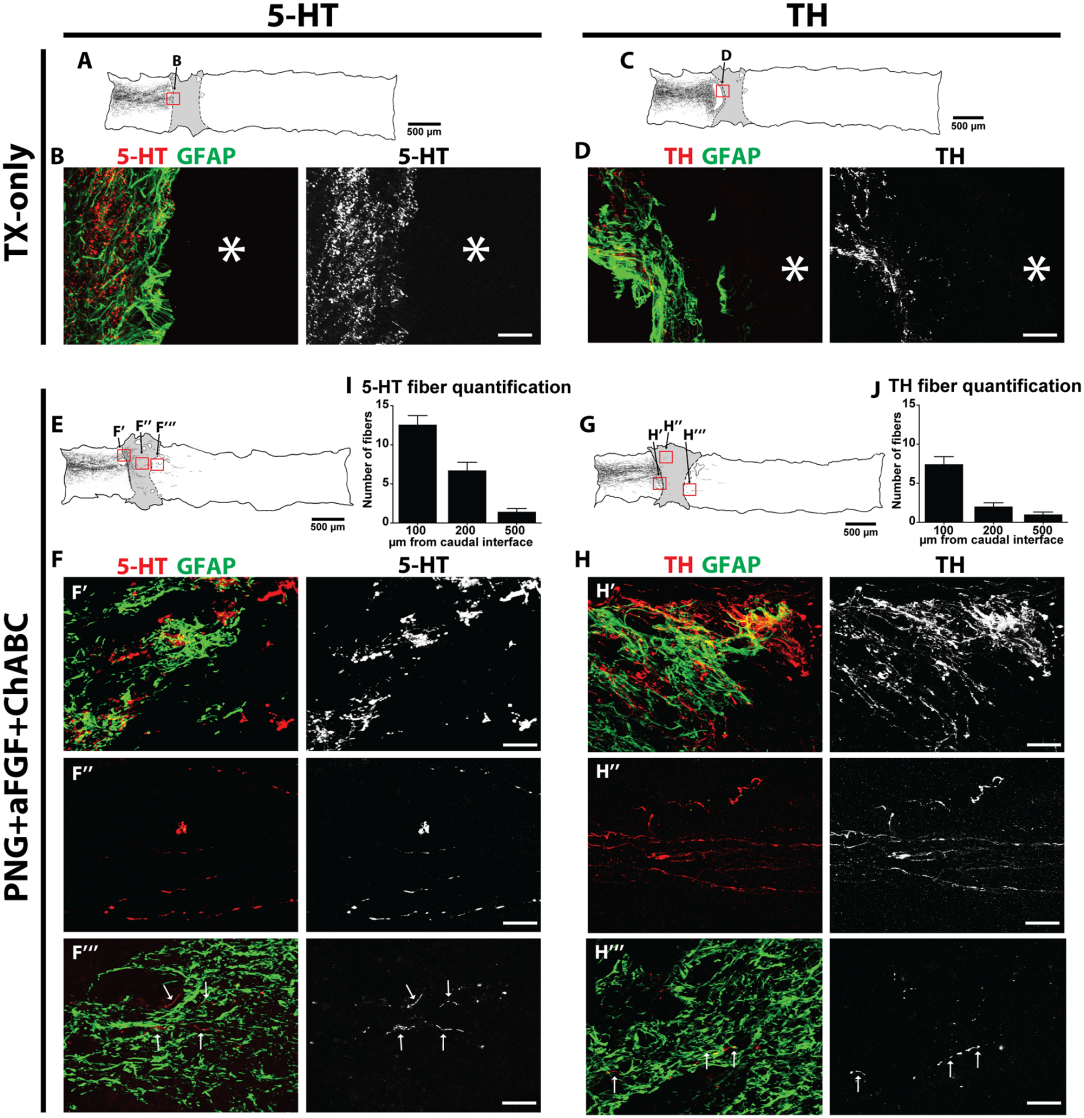
PNG+aFGF+ChABC treatment induces 5-HT and TH regeneration 18 weeks post-complete spinal cord transection. TX-Only: (A) Camera lucida tracing of 5-HT immunoreactive fibers in a TX-only animal. (B) 5-HT and GFAP immunostaining of the boxed area in (A), scale bar, 50 μm. (C) Camera lucida tracing of TH-immunoreactive fibers in a TX-only animal. (D) TH and GFAP immunostaining of the boxed area in (C). Scale bar, 50 μm. PNG+aFGF+ChABC: (E) Camera lucida tracing of 5-HT-immunoreactive fibers in PNG+aFGF+ChABC. (F) 5-HT and GFAP immunostaining of boxed areas in (E): (F’) Rostral spinal cord-PNG interface; (F”) within the PNG; (F”‘) caudal spinal cord. Scale bar, 50 μm. (G) Camera lucida tracing of TH-immunoreactive fibers in a PNG+aFGF+ChABC-treated animal. (H) TH and GFAP immunostaining of boxed areas in (G): (H’) Rostral spinal cord-PNG interface; (H”) within the PNG; (H”‘) caudal spinal cord. Scale bar, 50 μm. (I) Quantification of 5-HT-immunoreactive fibers found in caudal spinal cord, n = 7. (J) Quantification of TH-immunoreactive fibers found in caudal spinal cord, n = 7. Only animals whose spinal cord was processed and cut sagittally were included in the analysis.

Given the voiding improvements in mice receiving PNG+aFGF+ChABC treatment and a reduction in collagen scarring, we next examined whether axonal regrowth extended beyond the caudal PNG-spinal cord interface. Regeneration into the bridge and well beyond the transection site in the rat model has been more extensively studied and has been found to be critical in the recovery of urinary function. In the mouse, immunostained 5-HT and TH axons were traced via camera lucida projections of consecutive serial parasagittal sections for the TX-only (Fig 4A and 4C) and PNG+aFGF+ChABC (Fig 4E and 4G) groups. 5-HT- and TH-positive fibers were identified only in the rostral penumbra of the lesion in the TX-only group. They did not penetrate deeply within the lesion nor did they ever regenerate beyond the lesion (Fig 4A–4D). In the PNG+aFGF+ChABC group, both 5-HT- (Fig 4E and 4F) and TH-positive fibers (Fig 4G and 4H) were visualized crossing the rostral spinal cord-PNG interface (Fig 4F’ and 4H’) to enter the PNG (Fig 4F”and 4H”). Importantly, some fibers continued into the distal caudal spinal cord (Fig 4F”and 4H”‘) beyond the caudal PNG-spinal cord interface. Quantitative analyses of fibers in the distal spinal cord confirmed the presence of regenerated 5-HT (Fig 4I) and TH (Fig 4J) fibers beyond the PNG.

**Fig 5:**
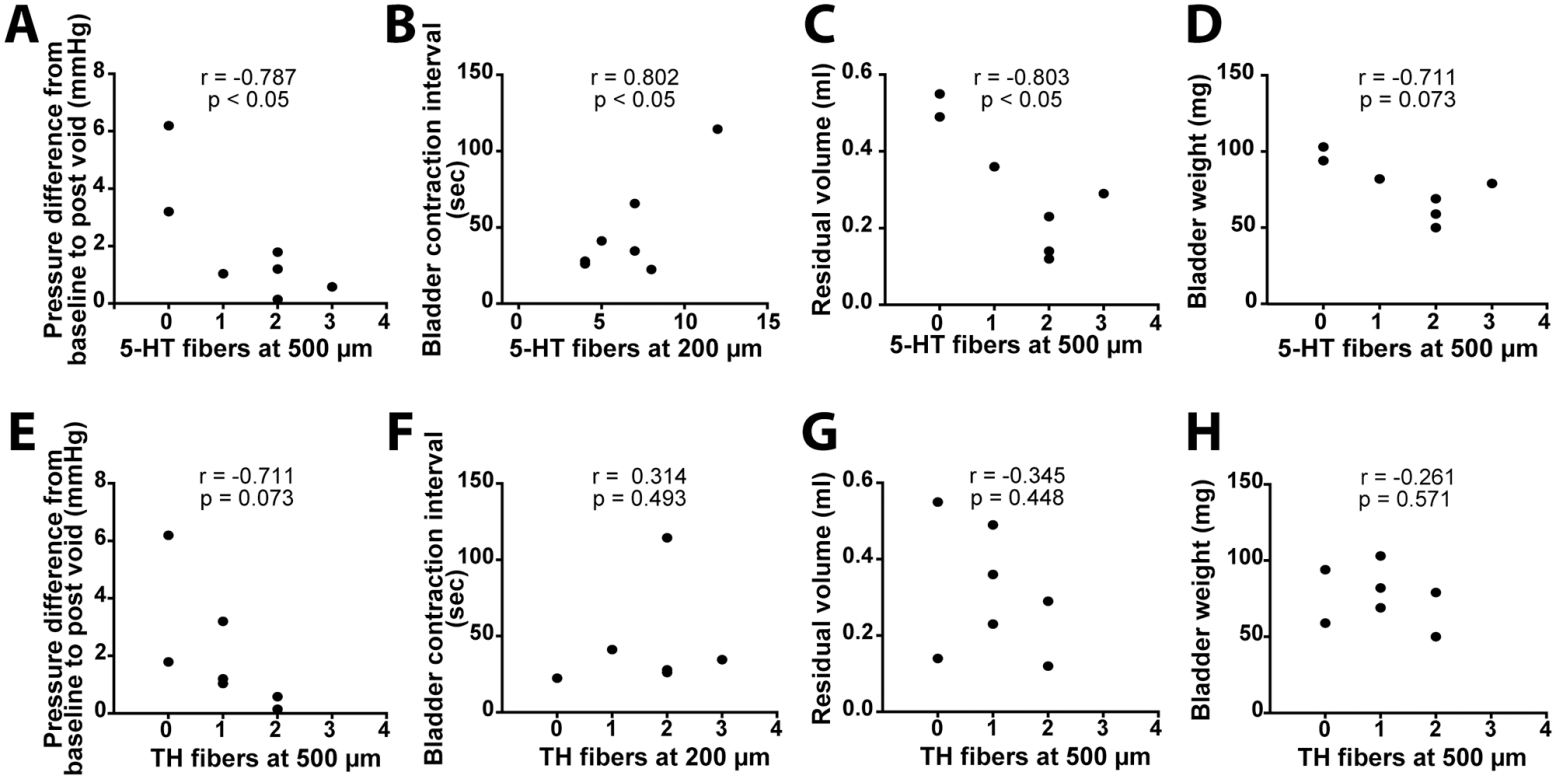
5-HT regeneration correlates to LUT function recovery. Pearson correlation coefficient (*r* value) comparing bladder recovery to the number of regenerating (A-D) 5-HT and (E-H) TH fibers found in the caudal cord 200μm or 500μm from the PNG-cord interface. The number of regenerated 5-HT fibers below the lesion site shows a significant correlation with the improvement of CMG parameters. There is no significant correlation between regeneration of the TH fibers and CMG parameter analysis. Only animals whose spinal cord was processed and cut sagittally were included in the analysis. n = 7.

Since both 5-HT and TH tracts mediate LUT recovery in the rat, we sought to correlate bladder recovery with regeneration in the mouse model. At the individual animal level, 5-HT regeneration was significantly correlated with an improvement of CMG parameters including the decreasing of pressure difference from the baseline to post void, the increasing of bladder contraction interval, and the decreasing of residual volume (Fig 5A–5D). No such correlation was found with TH regeneration, however, the baseline pressure following a void showed a non-significant trend (Fig 5E–5H). These results suggest 5-HT regeneration may be more important than TH regeneration for bladder recovery in a mouse model of SCI.

## Discussion

### Overview of results

The results of our investigation demonstrate the feasibility of employing the PNG+aFGF+ChABC combinatorial repair strategy in mice. These results corroborate our previous work [8] demonstrating that the triple combination treatment leads to improvements in voiding patterns, diminished detrusor sphincter dyssynergia, enhanced voiding efficiency, and improved bladder and EUS morphology. We also demonstrated a reduction in collagen scarring and regeneration of 5-HT and TH neurons through the peripheral nerve graft into the caudal cord. Our results suggest that 5-HT regeneration is more important for LUT recovery in the mouse than TH regeneration. In the rat, 5-HT innervation of the dorsal lateral nucleus of the lumbosacral spinal cord is required for the induction of EUS bursting activity during a void, and regeneration of the 5-HT tract is required for meaningful LUT recovery [26]. Conversely, silent periods or low EUS activity periods are important for efficient voiding in the mouse, and serotonin’s contribution to LUT recovery in a mouse model of SCI is not well understood. Nonetheless 5-HT regeneration is most likely needed for recovery in both models. The presence of 5-HT fibers distal to the lesion may also contribute to the preservation of the EUS muscle integrity by increasing the excitability of and synaptic input to motor neurons [27], as we have previously reported that PNGs enhance 5-HT regeneration and preservation of muscle mass and the myosin heavy chain phenotype following complete spinal cord injury [28]. Our previous rat study showed that TH regeneration is also required for efficient voiding, however, we found only trending correlation of TH regeneration and LUT function in a single parameter in the mouse model of SCI. Further, it is possible that chondroitinase and aFGF could be sufficient without the need of a graft to realize benefit by facilitating remodeling of intact spinal circuits. While the regeneration of 5-HT and TH fibers or other descending systems (not identified in the current study) likely contributes to bladder functional recovery, the precise role of frank regeneration in improved LUT behavior in the mouse remains to be determined by further investigation.

Although improvements in LUT recovery and axonal regeneration were evident with our triple combination therapy, the mouse model did not show the same strong nerve regeneration effects in both the 5-HT and TH systems as our previous rat study, in terms of either amount and distance [8]. This could be due to a variety of factors. First, the interface between the PNG and host tissues in the present study was not as striking as in our previous study in rats [8]. Second, mice, like humans, never develop a spinal reflex of urination after injury, while rats do. Sporadic recovery in the rat may prime the bladder circuitry such that with axonal regeneration, greater recovery is possible. Third, the pathology of the mouse SCI may perturb axonal regeneration. A mouse spinal cord lesion fills up with a dense fibrous connective tissue [15] rich in collagen which can act as a physical barrier to regeneration, while the transected rat spinal cord exhibited little collagen scarring. Lastly, due to the small size of the mouse, there are technical difficulties associated with placing a compressive S-shaped mono-filament surgical steel wire to stabilize the vertebral column in a dorsiflexed position, as we were able to do in the rat study. In rats, failure to stabilize the cord resulted in hindered regeneration [29]. Although it is likely the regenerating 5-HT and TH neurons failed to reinnervate their original target, there is considerable evidence that relay circuits can confer functional benefits distant from regenerating or injury induced sprouting tracts. For example, in the transected cord of the zebra fish, 5-HT and TH axons regenerate but fail to directly reinnervate their original motor neuron targets, however full recovery of swimming capability is achieved [30]. Similar observations have been made in the cortical spinal tract of primates, which normally have monosynaptic innervation onto motor neurons. Following transection of the corticomotoneuronal pathway, a disynaptic network forms restoring partial motor recovery [31].

### PNGs create a favorable regenerative environment

The concept of engrafting tissue or cells into an SCI lesion site to create a permissive environment for axonal regeneration has been proposed for almost a century [32]. Many different types of tissues or cells have been used, with varying levels of success, including Schwann cells [33], olfactory ensheathing cells [34,35], bone marrow-derived mononuclear cells [36], astrocytes [37,38], and various types of stem or progenitor cells [39–42]. To date, the application of the autologous peripheral nerve graft has been successfully used in animal studies [23,24,43–47] and even in human patients with SCI [48,49]. A PNG bridge provides a permissive, directional substrate to encourage and guide long-distance regeneration, and resident Schwann cells provide trophic support and can remyelinate the axons. Axonal growth into and along the PNG is robust, but most axons fail to exit the distal end of the graft. Expectedly, in studies using only a PNG, there is no behavioral improvement. In a primate model of SCI, transplantation of PNGs led to similar levels of axonal regeneration into the graft as seen in rodent studies [46]. Although no improvements in behavior were observed, this study demonstrated the capacity for primate CNS axons to grow into a PNG. These studies demonstrate a reproducible technique to regenerate axons past a lesion site. The challenges that lay ahead are to promote the re-entry of axons at the distal end of the graft back into the spinal cord.

### aFGF contributions to regeneration

Addition of growth factors to the PNG/cord interface, namely aFGF, can help bring about meaningful, physiologically-relevant regeneration [23,24,50]. aFGF can act through various mechanisms to enhance regeneration, as it interacts with many different FGF receptors and cells [51]. Several studies have identified FGF’s role in the reduction of collagen related scar formation and in down regulation of collagen gene expression in fibroblast [52–54]. Our study showed that PNG+aFGF+ChABC treatment reduced the collagen deposit which may be attributed to the effect of FGF application. It has been shown frequently that aFGF reduces axonal dieback and promotes regeneration thorough a graft [23,24,50,55]. Failure to include aFGF in a PNG or Schwann cell graft significantly diminishes the number of regenerating axons found within the graft [8,56]. aFGF may also be acting to decrease astrocyte activation [57] and to promote astrocyte migration and morphogenesis [8,58], encouraging better integration between the PNG and spinal cord. However, the aligned bipolar morphology of astrocytes between PNG and host tissues in this mouse model was not as obvious as in our previous rat study [8]. This can be attributed to more fibrotic scarring at the interface. Other trophic factors such as GDNF [59] can also be considered to further improve the interface.

### CSPGs impair axonal regeneration

A major impedance to regenerating axons following SCI is the upregulation of inhibitory CSPGs in the extracellular matrix. Digestion of CSPG side chains with the bacterial enzyme ChABC enhances axonal regeneration and sprouting in a variety of injury models [37,47,60,61]. The digestion of CSPGs also promotes the migration and integration of Schwann cells from the PNG into the host spinal cord [62] and aligns astrocytes towards the injury site [63]. Additionally, CSPG degradation of the perineuronal net distal to the graft could promote remodeling of spared pathways and integration of regenerating fibers into spared circuits [64]. Chondroitinase may also be modulating macrophage activation towards a more permissive phenotype [65], which has been shown to enhance axonal elongation in a CSPG-rich environment [66]. Failure to use chondroitinase in a PNG repair model of SCI results in very few axons leaving the graft/chord interface and causes diminished recovery [8,60].

## Conclusions

The current study suggests that peripheral nerve autografts supplemented with aFGF and chondroitinase support meaningful recovery of the lower urinary tract. This combinatorial therapy has shown efficacy in a number of different animal models and provides support for the need to target multiple modalities in the treatment of severe spinal cord injury.

## Supporting Information

S1 Fig A comparison of collagen distribution in mouse and rat after complete spinal cord transection. Representative photomicrographs of the spinal cord lesion stained with Masson’s trichrome from the transected mouse cord at 18 weeks post injury or the transected rat cord at 16 weeks post injury. Scale bar, 200μm for the mouse, 1mm for the rat. Left is rostral and right is caudal.
(TIF)
