# Supplementary figures and images for: Peripheral Nerve Transplantation Combined with Acidic Fibroblast Growth Factor and Chondroitinase Induces Regeneration and Improves Urinary Function in Complete Spinal Cord Transected Adult Mice
Source: PLoS One. 2015 Oct 1;10(10):e0139335. doi: 10.1371/journal.pone.0139335 (PMC4591338; doi:10.1371/journal.pone.0139335)

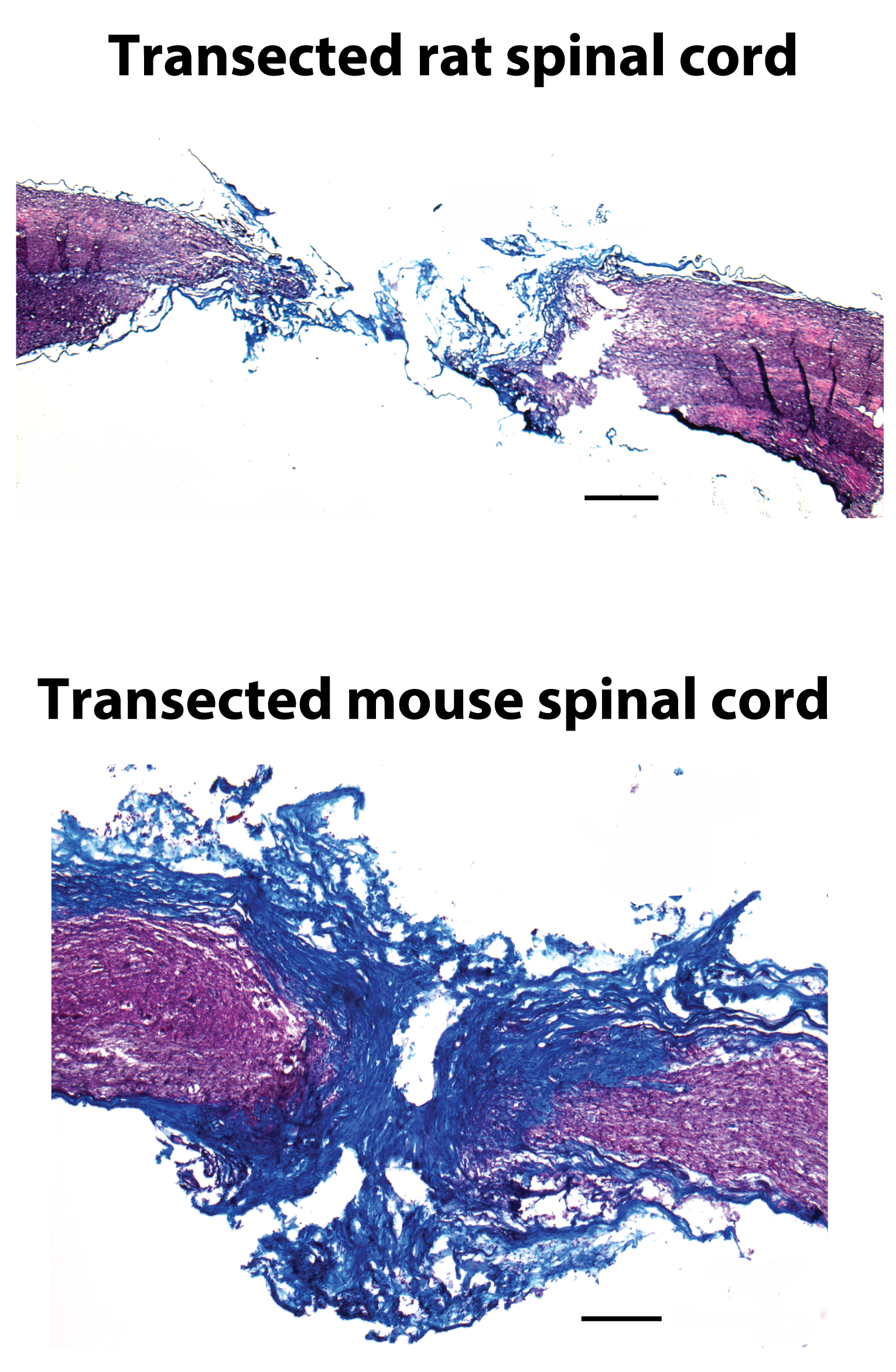

Supplement: S1 Fig — Representative photomicrographs of the spinal cord lesion stained with Masson’s trichrome from the transected mouse cord at 18 weeks post injury or the transected rat cord at 16 weeks post injury. Scale bar, 200μm for the mouse, 1mm for the rat. Left is rostral and right is caudal. (TIF) [file pone.0139335.s001.tif]
